# Under pressure – The working situation of Swedish healthcare managers during the first wave of COVID-19

**DOI:** 10.3389/fpsyg.2022.1052382

**Published:** 2023-01-11

**Authors:** Lisa Björk, Linda Corin, Magnus Akerstrom, Ingibjörg H. Jonsdottir, Alessio Degl Innocenti, Helle Wijk, Linda Ahlstrom

**Affiliations:** ^1^Region Västra Götaland, Institute of Stress Medicine, Gothenburg, Sweden; ^2^Department of Sociology and Work Science, University of Gothenburg, Gothenburg, Sweden; ^3^School of Public Health and Community Medicine, Institute of Medicine, Sahlgrenska Academy, University of Gothenburg, Gothenburg, Sweden; ^4^Department of Psychiatry and Neurochemistry, Centre for Ethics, Law and Mental Health (CELAM), Institute of Neuroscience and Physiology, Sahlgrenska Academy, University of Gothenburg, Gothenburg, Sweden; ^5^Region Västra Götaland, Sahlgrenska University Hospital, Gothia Forum for Clinical Trials, Gothenburg, Sweden; ^6^Institute of Health and Care Sciences, Sahlgrenska Academy, University of Gothenburg, Gothenburg, Sweden; ^7^Department of Quality Strategies, Region Västra Götaland, Sahlgrenska University Hospital, Gothenburg, Sweden; ^8^Department of Architecture and Civil Engineering, Chalmers University of Technology, Gothenburg, Sweden; ^9^Department of Orthopedics, Region Västra Götaland, Sahlgrenska University Hospital, Gothenburg, Sweden

**Keywords:** COVID-19 pandemic, health care managers, working conditions, job demands, job resources, Sweden

## Abstract

**Introduction:**

The aim of this study is to provide insight into the psychosocial work situation of hospital managers during the first wave of the COVID-19 pandemic.

**Methods:**

Mixed-effect modelling was used on survey data on job demands, job resources, job motivation, and work-life balance among over 500 managers working in 55 departments of a large Swedish university hospital in 2019 and 2020. Responses from 6011 employees were then used to stratify the analysis for COVID-19 exposure. Inductive content analysis was applied to open-ended questions on the managers’ views on organisational prerequisites during the onset of the pandemic.

**Results:**

The proportion of managers reporting difficulties with role clarity, quantitative demands, decision-making authority, and emotional support, time for recovery at work, motivation deficits, or problems with work-life balance clearly increased during the first wave of the pandemic. The proportion of managers reporting negative responses was higher in departments with high COVID-19 exposure. The qualitative analysis shows that overall governance in terms of clear, fair, and well-communicated routines, resource allocation, and division of responsibilities constituted an important framework for managerial during the crisis. First-line managers also require a mandate to re-organize their roles and their teams to successfully adapt to the situation. Organisational and social support was also important resources.

**Discussion:**

This is the first study investigating healthcare managers’ work situation during the first wave of the COVID-19 pandemic in a Swedish context. As expected, it indicates an increasingly strained work situation during the crisis, but it also provides findings on organisational prerequisites that allow healthcare managers to cope with stressful situations. In line with previous research on organisational resilience, the study provides suggestions for how higher-level managers can act in order to provide front-line managers with the organisational prerequisites they need to adapt, learn and develop successfully during times of unpredictability, insecurity, and rapid change in order to offer the best possible support to health care workers.

## 1. Introduction

The COVID-19 pandemic has placed healthcare providers under immense physiological and psychological pressures. The focus of previous research has been on the pandemic’s impact on frontline health care workers (HCW), revealing high workloads and mental health effects (Cabarkapa et al., 2020; Chersich et al., 2020; Demartini et al., 2020; Salazar de Pablo et al., 2020; Vizheh et al., 2020; Busch et al., 2021). The pandemic has thus magnified psychosocial risk factors in health care work (Theorell, 2020). Studies both on the effects of the COVID-19 pandemic and other epidemics on HCWs’ work situation and health point to specific needs among this group (Kisely et al., 2020; Billings et al., 2021; Busch et al., 2021). These needs include access to clear and concise information, disease-specific training, professional and emotional support, reliable access to adequate personal protection equipment, suitable working hours that enable recovery during and between shifts, and mental health screening with access to interventions for those in need. The responsibility for designing and implementing these protective measures has largely fallen on health care managers (HCM; Greenberg and Tracy, 2020; Kisely et al., 2020; Billings et al., 2021).

Managers are responsible for establishing an overview, stay focused, be positive, as well as monitoring employee health, especially during a pandemic (Bookey-Bassett et al., 2020; Theorell, 2020; Sihvola et al., 2022). Additional managerial tasks during a pandemic include the management of transfers of HCWs between departments, monitoring employees’ fear of infection, communicating ever-changing work routines and providing support in daily operations (Jonsdottir et al., 2021; Akerstrom et al., 2022). Thus, during periods when health care organizations are put under stress, the need for organizational structures that support managers in their role as leaders is intensified. Frontline nurse managers have for instance highlighted the need to be prepared for a crisis, and to adapt to constant changes of procedure (Vázquez-Calatayud et al., 2022).

Even before the pandemic, organizational changes, economic constraints and sub-optimal psychosocial working conditions, i.e., an imbalance between job demands and job resources, characterized managerial work in the health care field in many parts of the globe (Kelliher and Parry, 2015; Bjerregård Madsen et al., 2016; Labrague et al., 2018), and Sweden is no exception (Johansson et al., 2013; Vinberg et al., 2015). The retention and recruitment of skilled managers has become an area of great concern for the Swedish public sector, especially within health care (Vinberg et al., 2015; Cregård and Corin, 2019). While studies from Italy and Canada indicate an even more troublesome work situation for HCMs during the pandemic (Jackson and Nowell, 2021; Giusino et al., 2022), the impacts of the pandemic on the work situation of Swedish HCMs are still largely unknown.

The Job Demands-Resources (JD-R) model poses a useful theoretical framework for capturing the facilitators and hinderances at work (see for example Demerouti et al., 2001 for an overview). The JD-R model is well-established and has been used to predict a large number of health and performance outcomes across different occupational contexts (Schaufeli and Taris, 2014; Bakker and Demerouti, 2017; Lesener et al., 2019) including public sector managers (see for example Berntson et al., 2012; Giusino et al., 2022). The model assumes that all job characteristics can be classified as either a job demand, i.e., “*negatively valued* physical, social, or organizational aspects of the job that require sustained physical or psychological effort and are therefore associated with certain physiological and psychological costs” or a job resource, i.e., “*positively valued* physical, social, or organizational aspects of the job that are functional in achieving work goals, reduce job demands, or stimulate personal growth and development” (Schaufeli and Taris, 2014). In the JD_R model it is suggested that the specific demands and resources are context specific and must be chosen in relation to the target of a study or intervention (e.g., Schaufeli and Taris, 2014; Bakker and Demerouti, 2017).

Thus, in order to strengthen health care organizations for future pandemics, the aim of this study is to provide insights into Swedish HMCs’ work situation during the first wave of the COVID-19 pandemic using the JD-R model as theoretical framework. This was done through a mixed-method approach, where we first assess changes in managers’ working conditions, job motivation and work-life balance during the pandemic at a large Swedish university hospital and then highlight the organizational prerequisites managers perceived as important during the first wave of the pandemic.

## 2. Materials and methods

### 2.1. Setting

The study was conducted at Sahlgrenska University Hospital, one of the largest university hospitals in Northern Europe. The hospital provides emergency and basic care for the 700,000 inhabitants of the Gothenburg region and offers highly specialized care for the 1.7 million inhabitants of West Sweden. Additionally, there are centers of excellence in which Sahlgrenska University Hospital is a national and international leader. During the pandemic, the hospital was one of the leading institutions providing intensive care for COVID-19 patients.

### 2.2. Data material

In October 2019, all 647 managers and 15,870 employees at the hospital were invited to participate in a web-based survey about their psychosocial working conditions in terms of job demands, job resources, job motivation and work-life balance. The survey was distributed to all managers and employees in collaboration with the hospital’s Human Resources department. The reason for the survey was to get an overview of the psychosocial work environment of the organization, in accordance with recommendations from the Swedish Work Environment Authority. In September 2020, after the first wave of the pandemic, the same population was invited to a follow-up survey. This survey also included three open-ended questions regarding managers’ experiences during the pandemic, the organizational prerequisites they saw as valuable and those that were lacking. They were asked to recall their experiences during the first wave of the pandemic, with spring of 2020 as the starting point.

In total, answers provided from 617 managers (95%) in 2019 and 473 managers and 6,011 employees (88 and 41%, respectively) in 2020^1^ were used for the analysis (Table 1). Participating managers were employed at 70 different departments at the hospital. The administrative departments (*n* = 17) generally had only one manager per department and were consequently grouped together into “hospital and departmental level administration,” resulting in a final sample of 55 different departments (Table 2).

**Table 1:** Description of the managerial study group in 2019 and 2020 by managerial position, age and gender.

Variable	Category	Managerial characteristics	
		2019	2020
Total number of respondents, *n*		617	473
Type of manager, *n* (%)	Strategic manager	19 (3)	57 (12)
	Operational manager	390 (63)	264 (56)
	Manager with limited managerial responsibility	208 (34)	95 (20)
	Uncategorizable managerial position		57 (12)
Age, years (%)	≤29	7 (1)	2 (0.5)
	30–39	72 (12)	55 (12)
	40–49	206 (34)	140 (30)
	50–59	244 (40)	192 (41)
	≥60	82 (13)	83 (18)
Gender, *n* (%)	Female	467 (76)	365 (77)
	Male	138 (22)	106 (22)
	Other/do not want to answer	11 (2)	2 (0.5)

**Table 2:** Average number of managers, employees and COVID-19 exposure for the 55 departments during the first wave of the pandemic.

Variable	Department characteristics				
	Mean	Median	Standard deviation	Minimum	Maximum
Managers (*n*)	8.6	8.0	6.6	2.0	46
Employees (*n*)	109	86	68	20	319
Percentage of employees caring for COVID-19 patients (%)	48	49	26	2.8	95

### 2.3. Measures

**Figure 1:**
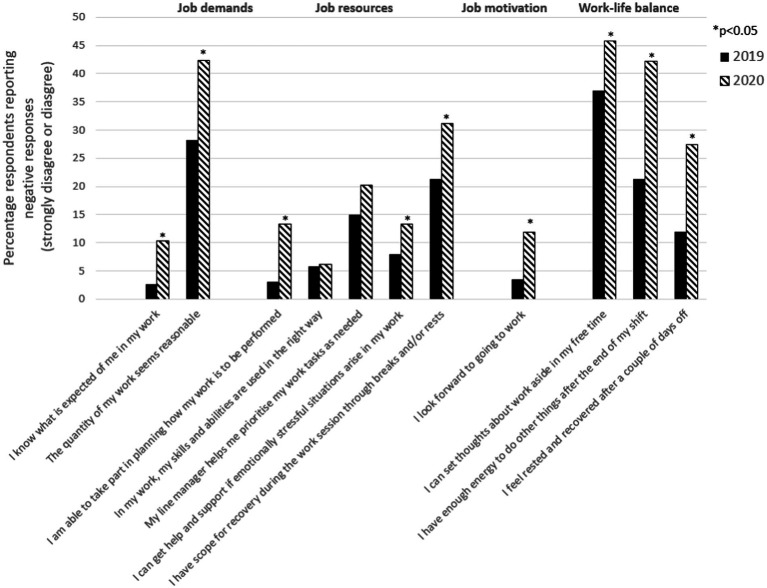
Changes in managers’ working conditions. The percentage of managers who strongly disagreed or disagreed with the statements in the survey regarding job demands, job resources, job motivation and work-life balance, thus reporting a negative situation before the pandemic (October 2019) compared with during the first wave of the pandemic (September 2020).

With the JD-R model as a conceptual framework, and inspired by Berthelsen et al. (2020) for operationalization, we used two variables to measure job demands (*Role clarity*, *Quantitative demands*) and five to measure job resources (*Decision-making authority*, *Skill discretion*, *Managerial support*, *Emotional support* and *Time for recovery at work*; Figure 1). The managers’ motivation was measured with a single indicator (*I look forward to going to work*). Work-life balance was captured using three indicators (*I can set thoughts about work aside in my free time*; *I have enough energy to do other things after the end of my shift*; *I feel rested and recovered after a couple of days off*).

In both surveys, all items were presented as statements with five response alternatives (*strongly agree*, *agree*, *neither agree nor disagree*, *disagree*, *and strongly disagree*). In the follow-up survey, managers also had the opportunity to provide information about important organizational prerequisites for conducting their managerial work during the pandemic. The baseline and follow-up surveys are described in detail in Jonsdottir et al. (2021).

The level of COVID-19 exposure for each department was determined using the percentage of employees reporting that they cared for patients with COVID-19 infection during the first wave of the pandemic in the spring of 2020. In order to investigate potential differences across departments and identify associations with the pandemic, the 55 departments were divided into three equally sized groups based on COVID-19 exposure (low, medium and high). In departments with *low exposure*, less than 32% of employees reported caring for infected patients (e.g., administrative departments, plastic surgery and rheumatology), while the proportions in the groups of *medium* and *high exposure* were 32–63% (e.g., cardiology, oncology and urology) and > 63% (e.g., infectious diseases department, emergency medical services and intensive care units), respectively.

### 2.4. Quantitative analysis

Changes in managers’ working conditions at the hospital and the variation in these changes across different departments were analyzed using the proportion of respondents in each department that disagreed or strongly disagreed with the statements in the two surveys. Mixed effects-models (Proc Mixed in SAS version 9.4; SAS Institute, Cary, NC, United States) were used with time (2019 or 2020 survey, nested within departments) as the fixed effect and information on departments as random effects (Akerstrom et al., 2021). Differences between departments were investigated by including an interaction term between the time and group variables. Hypothesis testing for fixed effects was performed using Wald tests, and tests of random effects were performed using likelihood ratio tests. Statistical significance was set at *p* < 0.05, and two-sided confidence intervals were used.

In a second step, the percentage of managers who strongly disagreed or disagreed with the statements in the survey regarding job demands, job resources, job motivation and work-life balance during the first wave of the pandemic were stratified for low, medium and high COVID- 19 exposure. Differences between these groups were tested using the mixed-effect models above, with a dummy variable for the exposure group added as a fixed effect on the second wave data only.

### 2.5. Qualitative analysis

The managers were given the opportunity to provide their own views on the organizational prerequisites that had been particularly important, or lacking, in their work during the first wave of the pandemic in spring 2020. Managers were also encouraged to share both positive and negative experiences from the COVID-19 pandemic. In all, the managers’ answers to these open-ended questions generated about 67 A4 pages of text. The first, second and third author performed a thorough reading of all written responses, followed by an inductive content analysis (Elo and Kyngäs, 2008) of about 10 pages for each author. The last author also read all of the material thoroughly and commented on the analyses. The suggested codes were compared and discussed, and codes were grouped into tentative categories. The remaining material was then coded, and categories were divided into overarching themes after discussions between the authors. The three themes were *Overall governance*, *Re-organization* and *Organizational and social support*.

### 2.6. Results from the quantitative analysis

The first research question concerned overall changes in managers’ working conditions between 2019 and the end of the first wave of the pandemic. When comparing the proportion of managers that were dissatisfied with their working conditions, job motivation and work-life balance at these two time points, a significant increase in negative responses was seen for nine of the eleven variables (Figure 1). In terms of job demands, the proportion of managers that disagree with the statement that they have *Role clarity* and a reasonable amount of *Quantitative demands* increased from 3 to 10%, and from 28 to 42%, respectively. When it comes to job resources, the proportion of managers that disagree with the statement that they have *Decision-making authority*, *Emotional support* and *Time for recovery at work* also increased significantly from 3 to 13%, 8 to 13%, and 21 to 31%, respectively. No statistically significant increase was found for *Skill discretion* or *Managerial support*. All three items of *Work-life balance* had a significant increase in negative responses; the proportion of managers disagreeing with the statements “*I can set thoughts about work aside in my free time*,” “*I have enough energy to do other things after the end of my shift”* and *“I feel rested and recovered after a couple of days off*” increased from 37 to 46%, 21 to 42%, and 12 to 27%, respectively. Lastly, the proportion reporting that they did not look forward going to work (i.e., *Motivation*) increased from 3to 12%.

**Figure 2:**
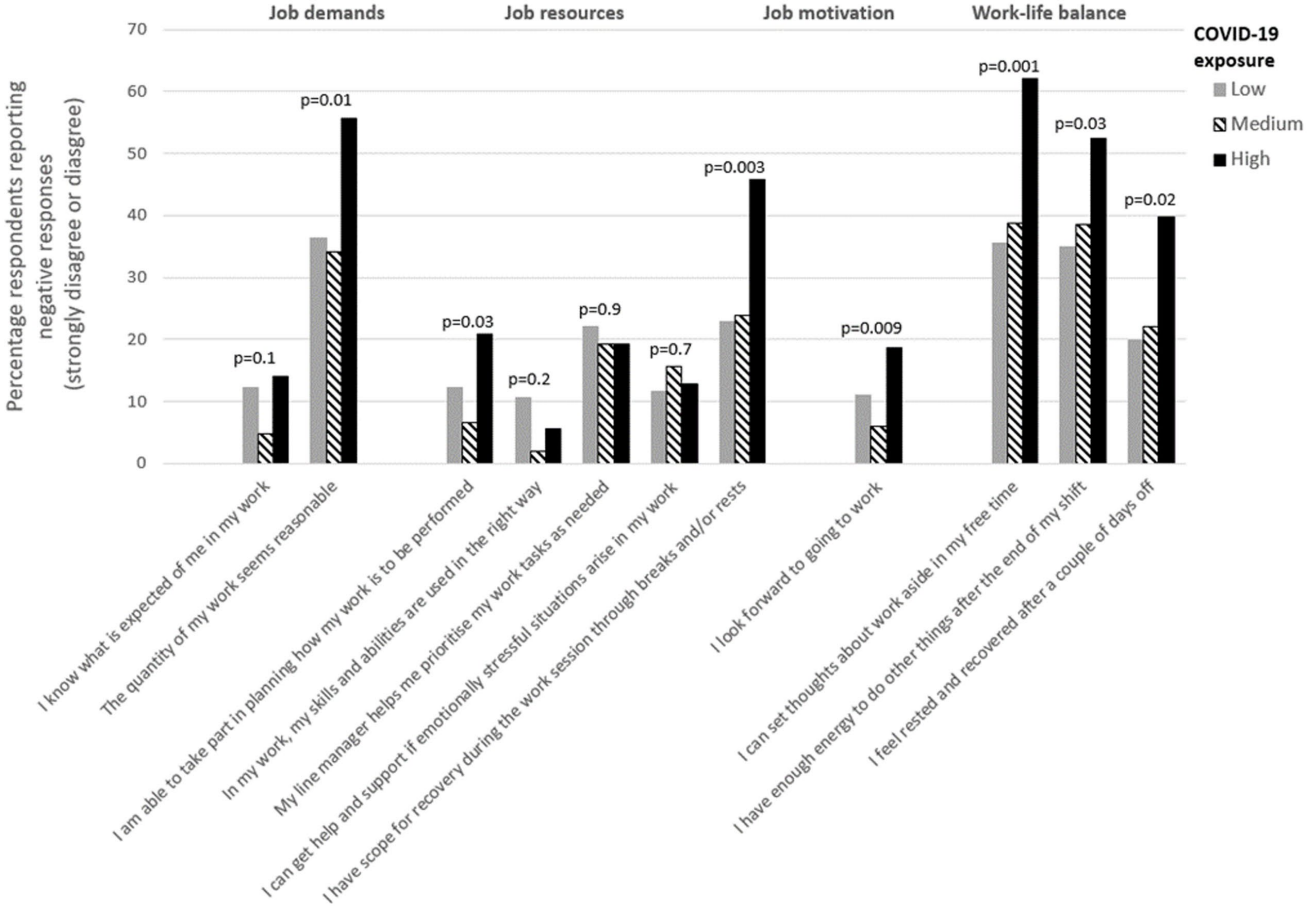
Managers reporting a negative situation during the first wave of the pandemic, stratified by COVID-19 exposure. The percentage of managers who strongly disagreed or disagreed with the statements in the survey regarding job demands, job resources, job motivation and work-life balance in the COVID-19 survey, thus reporting a negative situation during the first wave of the pandemic (in spring 2020), stratified by COVID-19 exposure group. Low, medium and high COVID-19 exposure at the department represents <32%, between 32 and 63% and >63% of the employees that cared for COVID-19 patients.

When investigating potential differences between departments, it was found that the managers’ working conditions differed significantly between the 55 departments at the hospital (*p* = 0.003 to <0.001 for all items). To further investigate the differences between departments and their association with the pandemic, we stratified the managers’ responses in 2020 depending on whether the department had low, medium or high COVID-19 exposure (Figure 2). The results show a statistically significant association between exposure and seven of the 11 items (*Quantitative demands*, *Decision-making authority*, *Time for recovery at work*, *Motivation* and the three variables within *Work life balance*), with a higher proportion of negative reports in the departments with high exposure compared to departments with low or medium exposure. No association between exposure and the variables *Role clarity*, *Skill discretion*, *Managerial support* and *Emotional support* was found.

## 3. Results from the qualitative analysis

To shed light on what organizational prerequisites managers perceived as important during the first wave of the pandemic, the results are presented following the three themes of *Overall governance*, *Re-organization* and *Organizational and social support* that emerged from the qualitative data.

### 3.1. Overall governance: Allocating resources, establishing routines and transferring responsibility

The way the hospital management team directed the organization through processes and routines was paramount for frontline managers’ work situation during the pandemic. Routines and guidelines established to protect the staff from the virus, for testing, for treatment of infected patients and handling the deceased changed constantly, often on a daily basis. Another important set of routines concerned staff planning and scheduling. Operative managers were responsible for lending out staff as well as receiving and introducing new staff to their units. And they were often required to shift their own work tasks, alternating between managerial responsibilities and serving in their roles as physicians or registered nurses. Furthermore, top-down strategies to re-allocate resources and decentralize decision-making authority to first-line managers were important measures taken to direct the organizations. Lower-level managers reported that being entrusted with decision-making authority to adapt workflows and work procedures to local conditions, without having to consult with upper management, was highly appreciated:

Mandate and room to manoeuvre to make decisions in order to be able to quickly adjust and adapt operations to rapidly changing circumstances. […] Clear orders/ clarity in how to prioritize as a basis for being able to quickly adapt activities.

Middle manager, Department of Psychiatry, Cognition and Geriatric Psychiatry, middle manager.

Top-down decisions on routines, resource allocation and the allocation of responsibilities were generally perceived as clear, fair and well communicated, thus serving as important frameworks for managerial action at the operational level:

The overall impression is that as a professional and manager, I had the freedom and space for action that I had never experienced before in my professional life.

First-line manager, Department of Anesthesia/Surgical Operations/Intensive Care.

As a head of department, I have benefited from a clear direction from the hospital management team, where we all affirmed what we knew and did not know about this pandemic.

Middle manager, Department of Obstetrics.

When top-down decisions and routines were perceived to be vague or unclear, poorly communicated or impossible to implement, this instead created a high degree of confusion and frustration, as well as feelings of organizational injustice:

Very frustrating to see guidelines on the website but not have access to protective equipment. Referring for a long time to visors (which we had to make ourselves) and plastic aprons as “safe” equipment.

Managerial level unknown, Department of Psychotic Disorders.

Initially, [I] experienced that there was huge ambiguity in decision-making issues. A lot took time when there was no time. The structure within the organization was wobbling.

First-line manager, Department of Anesthesia/Surgical Operations/Intensive Care.

Many managers were able to tolerate vague routines and an unclear division of authority in the beginning of the pandemic, but as time passed, they became less tolerant:

I needed clearer leadership, more support within the management team, that everyone helped staff the COVID-19 intensive care units to reduce the extremely heavy load some employees were exposed to, while others worked on as if nothing had happened. A shared responsibility among managers. Not that some did as they pleased and made their own decisions. Incredibly messy in the decision-making process, staffing requirements from different units…

First-line manager, Department of Anesthesia/Surgical Operations/Intensive Care.

Higher-level management, both at the regional and at the hospital level, used different strategies in communicating their decisions to lower-level managers. The COVID-19 website on the intranet was an important source of information. Here, all information relevant to HCWs was gathered, and the attached newsletter was updated regularly with changes to this information. The informants also refer to the ‘managerial newsletter’ that was sent by e-mail to all hospital managers, sometimes several times a day, as a key resource:

Very good to have daily managerial newsletters […]; they were clear and contained essential information. It made it easier to take my managerial responsibilities and inform the staff about the guidelines. We had daily morning meetings and brought up what was new for the day, which was needed because decisions and guidelines changed from day to day.

First-line manager, Department of Hybrid and Interventional Procedures.

However, some informants found the information flow overwhelming, both in terms of content and frequency, and sometimes even contradictory:

Extreme loads of different/updated information and routines initially; protective equipment, cleaning routines, routines in the event of death, etc. Various information channels […]. It became much easier when that sort of things settled down after a few weeks.

First-line manager, Department of Infectious Diseases.

### 3.2. Reorganizing to meet the needs of the COVID-19 pandemic

A key factor for managers’ ability to handle the demands of the pandemic was their ability to reshape both internal and external organizational structures. In many examples, respondents report that management teams met more frequently, used new digital techniques (often online meetings) and involved temporary members in order to facilitate decision making:

That we got a clear management structure where the roles were clear to everyone. A head of department with whom I had daily communication about most things and supported me. Good communication with other managers in other units who had their staff on loan to us. A section leader during the daytime throughout the COVID-19 crisis, where we worked closely together and knew who did what.

First-line manager, Department of Anesthesia/Surgical Operations/Intensive Care.

Many managers reported that their involvement in decision-making processes increased:

A common approach at the department level, clear information channels, participation in planning and design processes and increased room to manoeuvre and try new things to make the best of situations that arose.

First-line manager, Department of Geriatrics.

There were special work teams that were established to deal with particular aspects of the COVID-19 pandemic; for example, working teams to secure access to personal protective equipment and other COVID-19 related material. Further, there were teams established to deal with urgent staffing and scheduling issues at the hospital wards and emergency departments. There are also examples of improved collaboration between hospitals within Sweden and abroad, including departments, units and professions, to address common problems:

In the event of disasters such as these, the framework falls and we must follow general guidelines. Working hours of 12–16 h per day, seven days a week during the first four weeks required focus to bring in resources, structure the schedule, create the best possible conditions in order to increase the security of our employees. Then it is not possible to think of ordinary frameworks. Internal conflicts must be kept away; it takes far too much energy from managers and employees!

First-line manager, Department of Hybrid and Interventional Procedures.

### 3.3. Organizational and social support

Many managers reported that they needed strength, motivation and the ability to take actions themselves, especially at the start of the pandemic. However, they were part of an intricate web of social relations, and the need for communication and social support between and within the hospital’s organizational levels clearly intensified during the pandemic. One healthcare manager described the importance of social support and trust during a pandemic:

Support for first-line managers is important so that they can support employees. This was a situation where we exposed ourselves and our employees to a risk of becoming infected with a potentially deadly virus at work, to save lives. […]. To handle all the reactions that arose in the employees and in ourselves… Trust is important in a high-risk situation and applies to all levels, from employees to operational managers and at the strategic level. The obligation to report on the consequences of decisions, and not only “obey blindly,” usually falls on the first-line managers. But it is also something that requires courage in a hierarchical decision-making structure, such as that of a crisis organization. So trust and support for first-line managers is an absolute necessity in future crises.

First-line manager, Department of Anesthesia/Surgical Operations/Intensive Care.

Many first-line managers reported that their contact with their immediate manager increased and intensified during the pandemic and expressed their gratitude towards their superiors for being present, easy to access, supportive and direct in their communication. Support from the immediate manager is often mentioned as a key prerequisite for lower level managers to be the support their staff needed:

We got a clear management structure where the roles were clear to everyone. A department manager with whom I had daily contact most of the time and who supported me. Good communication with other managers in other units who had their staff on loan to us.

First-line manager, Department of Anesthesia/Surgical Operations/Intensive Care.

When there was a lack of managerial support, operative managers found themselves in vulnerable situations and reported feeling tremendous pressure to be strong, make decisions and take on great responsibilities with consequences for both themselves and their subordinates:

[It was difficult] to force employees to do a job that they felt very bad about, to have crying employees daily who asked to not have to go to the COVID 19 intensive care unit. But despite appealing to my manager and HR, I had no choice but to continue sending them there, even though they had anxiety, sleeping problems stomach pains, etc.

First- line manager, Department of Anesthesia/Surgical Operations/Intensive Care.

Managers expressed a second important source of social support, which originated from managerial colleagues. Daily management team meetings and informal contacts with managerial peers both provided a platform for sharing knowledge, making quick decisions, and coordinating and reallocating resources. Being a part of a managerial team also brought emotional support, providing the comfort and security needed in stressful and confusing situations:

The management team had daily meetings, and in that way, I got good support from my colleagues and a sense of security that we helped each other think about important issues.

First-line manager, Department of Psychiatry, Cognition and Geriatric Psychiatry.

At the beginning, it was quite messy at the hospital, before routines were established, and sometimes a division of responsibilities was lacking. The most important thing during the spring was our strong management team. We had to come up with solutions in our own way and make the contacts we needed to secure the care we provided.

Middle manager, Department of Nephrology.

Many informants tried to prioritize their physical presence among employees during the pandemic, and pointed to the registered nurses, assistant nurses, physicians and support functions as the key resources to combat the COVID-19 crisis. The importance of being able to rely on employees to go above and beyond for their patients and for the organization as a whole in an extreme situation cannot be overestimated. It provided a source of social support for first level managers to work together as a team and share responsibility for the difficult work that needed to be done:

[It was a positive experience] to see that you can do more than you think. To feel proud of the staff's flexibility. Most just did what was required, and that was no small task.... The sense of belonging... The staff’s commitment and an ability to find solutions…

First-line manager, Department of Orthopedics.

Dealing with concerned employees who feared catching the highly contagious virus, both to protect their own health and the health of family members, was also reported to be a challenge. Furthermore, dealing with perceived injustices when some employees were willing to take on a lot of responsibility and extra work while others were not, or having to force employees to work extra shifts and not allowing staff to take leave, were also seen as frustrating among managers:

What took the most time and energy was facing strong anxiety, especially among the immediate employees, where the anxiety was an obstacle to functioning in their professional role […]. I also had to perform some of the employees' tasks.

First-line manager, Department of Nephrology.

A last source of social support that was frequently mentioned among respondents was the relief offered by organizational support functions. Specialists in the HR, IT, communications and financial services departments assisted the managers in their daily work in issues such as recruitment, legal matters, purchases and financial planning. For example, HR tasks, such as staffing and scheduling, changed character during the pandemic and would have become both complicated and time-consuming for managers to deal with on their own.

Respondents expressed special appreciation for the care hygiene unit, which supported, guided and advised other units, managers and individuals to prevent themselves and patients from being infected with the virus.

The ability of these functions to be flexible and responsive to sudden operational needs was a necessity and highly appreciated by the managers:

Those of us who work close to the patient have gained somewhat more influence than before. Administrative staff have worked to support us. In normal cases, it is usually the health care staff who are engaged by the administrative staff.

Team-leader, Department of Neurological Care.

Some things that were previously difficult to implement and call attention to were suddenly done quickly (when the will obviously appeared in the right person / function). This particularly applies to challenges in ICT.

Middle manager, Department of Clinical Microbiology.

In cases where the support functions instead failed to adapt to the situation caused by COVID-19, when they were seen as distant or stuck in ‘business as usual’, managers felt abandoned, stressed or exhausted:

It could have been organized so that a lot of administration, such as public transport cards, introduction of new systems, etc. could be tasks for administrators and HR. Support for managers was lacking in general. We had an extremely high workload, both first-line managers as well as section managers and operations managers.

Middle manager, Department of Oncology.

## 4. Discussion

This is, to our knowledge, the first study investigating HCMs´ work situation during the first wave of the COVID-19 pandemic in a Swedish context. The overall picture that appears from the analysis is that the vast majority of managers were satisfied with their work environment, felt motivated and reported acceptable levels of work-life balance both before and during the first wave of the pandemic. The proportion of managers reporting dissatisfaction with working conditions, motivation or work-life balance varied between 3% (for *Role clarity*, *Decision authority* and *Motivation*) and 37% (for the first indicator of work-life balance) in 2019. Not surprisingly, however, the results show that the situation deteriorated during the first wave of the pandemic. The proportion of managers reporting difficulties with *Role clarity*, *Quantitative demands*, *Decision-making authority*, *Emotional support* and *Time for recovery at work*, lack of motivation or problems maintaining a work-life balance clearly increased during the first wave of the pandemic. Particularly, *Quantitative demands* and the proportion of managers disagreeing with the statements ‘*I have enough energy to do other things after the end of my shift’* and *‘I feel rested and recovered after a couple of days off*’ increased between 2019 and the first wave of the pandemic. Together, these findings indicate an increasingly strained work situation for managers during the pandemic. These results are also in line with the JD-R theory, where an increased imbalance between job demands and job resources, and thus strained work situation, renders unwanted outcomes such as reduced motivation and work-life balance (see for example Bakker et al., 2014; Schaufeli and Taris, 2014).

However, no significant changes in *Skill discretion* and *Managerial support* were found.

The results show that the changes in working conditions varied between departments at the hospital. When investigating the association between managers’ working conditions and COVID-19 exposure at the department level, it was found that for seven out of eleven variables, the proportion of managers reporting negative responses was higher in departments with high exposure to COVID-19. No such association was found for *Role clarity*, *Skill discretion*, *Managerial support* and *Emotional support*. Thus, no association was found between these four working conditions and the degree to which employees at the respective departments cared for COVID-19 patients.

One obvious impact from the pandemic was an increased workload, not only among HCWs but also HCMs: the amount of work increased while time allocated for recovery diminished, as was also seen in other countries (Jackson and Nowell, 2021; Giusino et al., 2022). This is likely an inevitable consequence of a pandemic in the health care sector. However, there are many things top-level managers can do to alleviate this work strain. With the help of a qualitative analysis of the answers to three open-ended questions in the COVID-19 survey, three important remedies were found. First, when top-down decisions on routines, resource allocation and division of responsibilities are perceived as clear, fair and well-communicated by the managers, *Overall governance* constitutes an important framework for managerial action at the operational level. Particularly important areas for top-down routines and guidelines include information about how to work with infection prevention and how to schedule and re-allocate hospital staff. Managers at the lower levels also need the mandate to adapt workflows and work procedures to local conditions and ever-changing preconditions. Second, first-line managers need the mandate to *re-organize their roles and their teams to deal with the pandemic*. More frequent team meetings, an increased use of digital techniques, more collaboration across departments and professional groups were common measures taken to increase involvement in important decision-making processes. Other studies have expressed the importance of the team regarding support in decision making. Involving the staff in the decision making helped create a feeling of solidarity between the workers and the managers and increased their sense of belonging during the health crisis (Beogo et al., 2022). The third theme illustrates the significance of *organizational and social support* from the immediate manager, managerial colleagues and support functions such as HR specialists, experts in infection medicine and care hygiene, and the communication department. The same experience was expressed among frontline nurse managers who tried to be role models, to keep calm and carry on (Vázquez-Calatayud et al., 2022), they encouraged healthcare staff to keep going despite the constant uncertainty and ambiguity (Bookey-Bassett et al., 2020). Our study is in line with a previous study showing that higher perception of organizational support was shown to minimize managers’ perception of being challenged in times of a pandemic (Gab Allah, 2021). Finally, the informants point to their employees as a key resource in their role as managers during the pandemic.

All of these factors are things that higher level management should facilitate to decrease the negative impact on lower-level managers’ working conditions, as seen in the present study and similar studies (Jackson and Nowell, 2021; Giusino et al., 2022). Securing sustainable working conditions and adequate decision latitude, where individuals can make decisions and exercise control over their work and offer support to lower-level managers, will also increase the opportunity for these managers to implement preventive measures targeted at the specific needs of HCWs, as identified in the COVID-19 pandemic and previous outbreaks (Greenberg and Tracy, 2020; Kisely et al., 2020; Billings et al., 2021).

An organization’s capacity to adapt, learn and develop during times of unpredictability, insecurity and rapid change is often referred to as organizational resilience. A resilient health care organization supports HCWs in foreseeing, adapting and recovering so that they can provide high quality care (Jeffcott et al., 2009; Rangachari and Woods, 2020) while being protected from negative work-related outcomes (Baskin and Bartlett, 2021). The ability to anticipate severe crises increases when organizations learn, develop, adapt and move forward after stressful events and crises. By focusing on assigning organizational capabilities and working on relationships and interactions between organizational actors in these different phases, resilient organizations create trust, empowerment and safety among individuals and teams (Duchek, 2020; Rangachari and Woods, 2020). The ability to recognize, confront and deal with harsh conditions, fear, frustration, uncertain environments and other difficulties related to a crisis is vital maintaining resilience in the future. In this way, an organization can undergo development and recovery and thrive at the individual, team and organizational level even after a period focused on mere survival. One way to be prepared for a crisis is to arrange disaster management courses to provide knowledge and confidence beforehand (see for example Cariaso-Sugay et al., 2021).

### 4.1. Methodological considerations and future research

The fact that individuals cannot be followed over time placed methodological constraints both on the current analysis and future analyses. In this study, managers’ working conditions, in terms of job demands and job resources, were measured at the department level, assuming that the respondents represent all managers from each department. However, there was a high response rate among managers: 88% in 2019 and 95% in 2020, thus limiting the risk of bias.

Another limitation is the relatively low response rate (41%) among employees. This data was used to group the departments into low, medium and high COVID-19 exposure groups, and it is plausible that the low response rate could affect the reliability of this exposure measure. However, after reviewing the distribution of departments into these three exposure groups, the low exposure group included all administrative and psychiatric departments as well as departments with technicians, while the high exposure group included all intensive care units, and infectious disease departments. Thus, the departments are distributed as expected according to their proximity to COVID-19 patients.

When measuring both the effect and the outcome in a single survey, common method bias is a cause of concern. In this study, the main outcomes are based on two separate surveys distributed before and during the pandemic (see for instance Figure 1), or on a combination of answers provided from the managers and their employees (see Figure 2), limiting the risk of common method bias. In addition, the analyses were performed on the workplace level, not at the individual level.

In Sweden, the majority of HCMs have a license as registered nurses, or licensed physicians. This clinical expertise legitimizes the HCMs’ managerial role in the eyes of the employees giving them authority, trustworthiness, and confidence (Doria, 2015; Bugajski et al., 2017). However, it is uncommon in Sweden to let nurse managers supervise physicians or vice versa. During COVID-19 many managers were transferred to clinical work as a way to allocate resources to the COVID-19 care units. Unfortunately we have no data on the managers’ profession, or on how common it was with clinical work during the study period. One major strength of this study was the availability of pre-pandemic measures of working conditions among managers. In autumn 2022, a third data collection will take place, and this will make it possible to investigate the long-term effects of the pandemic on all HCWs at the hospital.

## 5. Conclusion

The findings of this study point to an increasingly strained work situation for health care managers during the first wave of the COVID-19 pandemic. In line with previous research on organizational resilience, the results also provide suggestions for how higher-level managers can act in order to provide front-line managers with the organizational prerequisites they need to adapt, learn and develop successfully during times of unpredictability, insecurity and rapid change in order to offer the best possible support to health care workers.

### 5.1. Practical implications

There is a variation in working conditions between departments at the hospital showing that the proportion of managers reporting poorer work conditions was higher in departments with high exposure to COVID-19. We provide several suggestions what can be done to alleviate this high work strain. First, top-down decisions on routines, resource allocation and division of responsibilities need to be perceived as clear, fair, and well-communicated. Particularly important areas include information about how to work with infection prevention and how to schedule and re-allocate hospital staff. Second, first-line managers need the mandate to make decision and re-organize and support their teams to deal with the pandemic. As such, more frequent team meetings, an increased use of digital support systems, more collaboration across departments and professional groups are some of the measures that can be used to enable managers to better encounter the situation and improve the decision-making processes. Third, support from different function should be organized including immediate manager, managerial colleagues and support functions such as HR specialists, experts in infection medicine and care hygiene, and the communication department.

## Data availability statement

The raw data supporting the conclusions of this article will be made available by the authors, without undue reservation.

## Ethics statement

The studies involving human participants were reviewed and approved by the Swedish Ethical Review Authority (ref. 2020-04771). The patients/participants provided their written informed consent to participate in this study.

## Author contributions

MA, IJ, AI, HW, and LA were responsible for data collection. MA performed the quantitative analyses in collaboration with LB and LC. LB, LC, MA, and LA conducted the qualitative analysis. LB, LC, and LA drafted the first version of the manuscript. All authors contributed to the final draft and read and approved the manuscript.

## Conflict of interest

The authors declare that the research was conducted in the absence of any commercial or financial relationships that could be construed as a potential conflict of interest.

## Publisher’s note

All claims expressed in this article are solely those of the authors and do not necessarily represent those of their affiliated organizations, or those of the publisher, the editors and the reviewers. Any product that may be evaluated in this article, or claim that may be made by its manufacturer, is not guaranteed or endorsed by the publisher.

## Footnotes

^1^After excluding 5 individuals who did not give their informed written consent.

## Abbreviations

HCW: Health care workers
HCM: Health care managers
HR: Human resources
IT: Information technology
